# Validation of the International Medullary Thyroid Cancer Grading System and Identification of EZH2 as a Prognostic and Potential Therapeutic Marker in Medullary Thyroid Cancer

**DOI:** 10.3390/cancers17050737

**Published:** 2025-02-21

**Authors:** Eline C. Jager, Bettien M. van Hemel, Bea Rutgers, Wouter T. Zandee, Liesbeth Jansen, Schelto Kruijff, Thera P. Links

**Affiliations:** 1 Department of Internal Medicine, Division of Endocrinology, University Medical Center Groningen, Hanzeplein 1, 9731 GZ Groningen, The Netherlands; e.c.jager@umcg.nl (E.C.J.);; 2 Department of Surgery, Division of Surgical Oncology, University Medical Center Groningen, Hanzeplein 1, 9731 GZ Groningen, The Netherlands; 3 Department of Pathology, University Medical Center Groningen, Hanzeplein 1, 9731 GZ Groningen, The Netherlands; 4 Department of Nuclear Medicine and Molecular Imaging, University Medical Center Groningen, Hanzeplein 1, 9731 GZ Groningen, The Netherlands; 5 Department of Molecular Medicine and Surgery, Karolinska Institutet, Solnavägen 1, 171 77 Stockholm, Sweden

**Keywords:** medullary thyroid cancer, EZH2, IMTCGS, biomarkers, prognosis

## Abstract

**Background:** Medullary thyroid cancer (MTC) is a heterogeneous disease. While the International MTC Grading System (IMTCGS) provides baseline risk stratification, it lacks therapeutic relevance. In several cancers, EZH2 overexpression harbors an adverse prognosis, with several EZH2 inhibitors undergoing investigation. This study validated the IMTCGS and examined the prognostic value of EZH2 and other biomarkers. **Methods:** Clinical data were collected and MTC specimens were retrospectively reviewed and morphologically assessed. Immunohistochemistry (IHC) of Ki-67 allowed IMTCGS validation. IHC of EZH2, PD-L1 and PSMA was evaluated on a tissue microarray (TMA). **Results:** Of 64 MTCs, the median tumor size was 28 mm (IQR 15–40). Coagulative necrosis, ≥5 mitoses, and Ki-67 ≥ 5% was seen in nineteen (30%), three (5%) and seven (11%) cases. Median Ki-67 was 0.9% (IQR 0.4–2.1). Forty-three (67%) and twenty-one (33%) were classified as IMTCGS low- and high-risk, respectively. High-risk tumors were associated with lower distant metastasis-free survival (DMFS) (HR 5.651, *p* = 0.017), locoregional recurrence-free survival (LRFS) (HR 18.323, *p* < 0.001) and disease-specific survival (DSS) (HR 10.001, *p* = 0.002), but not with overall survival (OS) (HR 2.109, *p* = 0.146). EZH2 expression was identified in 39/46 (85%) cases on the TMA. An expression of ≥10% (9/46, 20%) was predictive for DMFS (HR 4.747, *p* = 0.030), LRFS (HR 4.242, 0.039), DSS (HR 19.736, *p* < 0.001) and OS (HR 8.386, *p* = 0.004). PD-L1 and PSMA had no prognostic value. **Conclusions:** This study validates the prognostic value of the IMTCGS and identifies EZH2 as a novel prognostic biomarker in MTC patients. The therapeutic potential of EZH2 warrants further investigation in larger cohorts.

## Simple Summary

Medullary thyroid cancer has long been recognized as an elusive malignancy characterized by significant disease heterogeneity. The recent introduction of the International Medullary Thyroid Cancer Grading System (IMTCGS) has substantially enhanced postoperative risk stratification. This study aimed to validate the IMTCGS within a Dutch cohort of patients with medullary thyroid cancer. Since the IMTCGS has no therapeutic implications, we also explored the immunohistochemical expression of the epigenetic regulator EZH2, which is a potential therapeutic target, for the first time. Our validation of the IMTCGS (64 patients) further supports its prognostic value. Of the 46 patients analyzed for EZH2 evaluation, 39/46 (85%) were positive for the marker. Notably, patients with expression levels > 10% (9/46, 20%) exhibited poorer clinical outcomes. These novel findings suggest that EZH2 may serve as a promising therapeutic target in selected patients with medullary thyroid cancer, potentially paving the way for new systemic treatment options with EZH2 inhibitors.

## 1. Introduction

Medullary thyroid carcinoma (MTC) is a rare neuroendocrine tumor that arises from the C-cells in the thyroid gland [1]. MTCs are known to be heterogeneous and elusive tumors with a highly variable clinical course. Even patients with extensive distant metastases may have a long and indolent disease course, surviving years after their initial diagnosis, while others progress shortly after diagnosis [2].

To predict the variability in disease progression and enhance baseline risk stratification, the genetic and histopathological characteristics of MTCs have been the subject of many studies [2,3,4]. Recently, the International Medullary Thyroid Cancer Grading System (IMTCGS) was established, which divides patients into low- and high-risk categories based on histopathology. High-risk tumors with coagulative necrosis and/or ≥5 mitoses per 2 mm^2^ and/or a proliferation index (Ki-67) ≥ 5% were found to have worse locoregional recurrence-free, distant metastasis-free, disease-specific survival and overall survival [5].

Analyses of genetic alterations involved in the oncogenic proliferation of MTC have revealed the Rearranged during Transfection (*RET*) gene as a proto-oncogene [6]. *RET* mutations are prevalent in sporadic MTCs and give rise to the hereditary Multiple Endocrine Neoplasia type 2 (MEN2) syndrome when mutated at the germline [7]. *RET* encodes a transmembrane tyrosine kinase receptor, RET, that regulates multiple cellular pathways. When RET is abnormally activated by genetic mutations, it triggers uncontrolled cellular proliferation, differentiation and survival, ultimately leading to the development of MTC [8].

In recent years, advances in epigenetics have revealed that human cancer cells exhibit widespread epigenetic abnormalities, in addition to genetic alterations. Epigenetic alterations may occur through aberrations in DNA methylation and histone modification patterns and the expression of non-coding RNA [9]. The potential reversibility of these epigenetic aberrations, in contrast to genetic mutations, may allow them to be restored to their normal state through epigenetic therapy, which has led to several phase I–II trials of epigenetic inhibitors [10].

Enhancer of Zest Homolog 2 (EZH2) is a histone methyltransferase and catalytic subunit of the Polycomb Repressive Complex 2 (PRC2) that plays a major role in transcriptional repression. EZH2 enables chromatin remodeling through trimethylation of Lysine-27 in histone 3 (referred to as H3K27me3). H3K27me3 is associated with gene suppression and considered a vital but potentially reversible epigenetic alteration [10,11]. Overexpression of EZH2 has been reported in various cancers, including breast cancer, esophageal cancer, poorly differentiated and anaplastic thyroid cancer, and several neuroendocrine cancers, where it seems to harbor an adverse prognosis [11,12,13,14,15]. One study has revealed potential overexpression of EZH2 in MTC at the genetic level [16]; however, to our knowledge, EZH2 protein expression in MTC tissue has not been evaluated.

In the current study, we performed a validation of the IMTCGS in a Dutch population of MTC patients. In addition, we performed an exploratory study to elucidate EZH2 protein expression in MTC tissue, as well as the expression of several other immunohistochemical markers with potential diagnostic or therapeutic value (prostate-specific membrane antigen [PSMA] and programmed death-ligand 1 [PD-L1]). Finally, we assessed their association with clinical outcomes.

## 2. Materials and Methods

### 2.1. Patients

This is a retrospective analysis of 64 MTC patients from the University Medical Center of Groningen (UMCG). All MTCs were confirmed on histopathology after thyroid surgery, which took place between 2000 and 2022. All primary MTC tumors with a ≥5 mm diameter and sufficient available tumor tissue were included. Through a retrospective review of patient files, data on diagnosis, treatment and follow-up were obtained. All patients were classified according to the 8th edition of the tumor-node-metastasis (TNM) classification and staged according to the criteria in the American Joint Committee on Cancer (AJCC) for MTC.

### 2.2. Definitions

Patients were deemed biochemically cured postoperatively and at final follow-up when their calcitonin concentrations were below the reference value. Calcitonin was assessed with an enzyme-linked immunoassay (Biomerica, Irvine, CA, USA) between 2002 and 2010 (reference: 0.3–12 ng/L), a chemiluminescent immunoassay (Diagnostics Products Corporation, Los Angeles, California, United States [later acquired by Siemens]) between 2011 and 2014 (reference: <18.2 for males and <11.5 for females) and an electrochemical illuminescent immunoassay (Roche, Basel, Switzerland) between 2014 and 2022 (reference: <10 ng/L). Distant metastasis (M1) was considered present at diagnosis if identified within six months of the initial thyroid surgery. Mediastinal lymphadenopathy (level 7) was considered part of the central compartment and not classified as M1. If locoregional structural evidence of disease appeared after six months, we considered these locoregional recurrences. Evidence of structural recurrent disease was based on imaging and/or pathology results.

### 2.3. Histopathology and Construction of Tissue Microarray

Surgically removed tumors underwent standard processing in the Department of Pathology of the UMCG. The tissue was formalin-fixed and paraffin-embedded (FFPE), and 4 μm sections were cut and placed on glass slides for digital image acquisition (described below). For morphological assessment, FFPE slides were stained with hematoxylin and eosin (HE).

In addition, a tissue microarray (TMA) that had been constructed for a previous study was used. The TMA was manually constructed by taking three cores of 0.6 mm, punched from tumor-rich areas in the FFPE blocks of the primary tumor. Of the 64 MTC patients, tissue cores from 46 patients were available on the TMA.

### 2.4. Digital Images

To allow digital review, glass slides were scanned using a Philips Ultra Fast Scanner 1.6 (Philips, Eindhoven, The Netherlands) equipped with a 40× magnification lens and employing a single focus layer without Z-stacking. Optimal image quality was ensured through automatic tissue detection with focus points. The digitized slides were then stored on a centralized image server.

### 2.5. Morphological Assessment

The primary tumors were independently reviewed by an expert thyroid pathologist (B.M.) and an experienced MTC researcher (E.J.), after which consensus was reached. The following parameters were assessed: tumor size, degree of capsulation (none, partially, completely), vascular invasion, perineural growth, extrathyroidal extension, tumor at the resection margin, multifocality and bilaterality. In addition, the presence of any visible growth pattern was scored (solid, lobular, trabecular, cribriform, follicular). Any visible cell shape was identified (polygonal, round, spindled morphology) and nuclei were assessed for their grade (low, intermediate, high) and the presence of multinucleated cells and prominent nucleoli. Since an evident distinction between amyloid and fibrosis deposition cannot be assessed without additional staining, the presence of fibrosis and amyloid was combined into one variable (fibrosis/amyloid: none, mild, moderate, prominent). In addition, the presence of microcalcifications was determined. Finally, the number of mitoses per 2 mm^2^ and the presence of coagulative necrosis were assessed, according to IMTCGS [5].

### 2.6. Immunohistochemistry

Immunohistochemistry (IHC) with CONFIRM anti-Ki-67 Clone 30-9 (rabbit monoclonal antibody, RTU, Ventana Medical Systems, Illkirch, France) was performed on 3 μm sections of whole tissue slides. IHC on 3 μm sections of the TMA was conducted using EZH2 Clone D2C9 (rabbit monoclonal antibody, Cell Signaling Technology, Danvers, MA, USA), PD-L1 Clone SP263 (monoclonal rabbit antibody, RTU, Ventana Medical Systems, Illkirch, France), PD-L1 Clone 22C3 (mouse monoclonal antibody, Dako/Agilent Technologies Netherlands B.V., Middelburg, The Netherlands), PSMA Clone 3E6 (mouse monoclonal antibody, DAKO/Agilent Technologies Netherlands B.V., Middelburg, The Netherlands) and CD31 Clone JC70 (mouse monoclonal antibody, RTU, Ventana Medical Systems, Illkirch, France). IHC for PD-L1, CD31 and Ki-67 was performed on a Ventana BenchMark Ultra immunostainer (Ventana Medical Systems, Illkirch, France). IHC of EZH2 was performed manually with microwave antigen retrieval with EDTA, pH 8.0, dilution of 1:50, 1 h incubation time, and detection with secondary antibody (goat anti-rabbit labeled with horseradish peroxidase, dilution of 1:100). Visualization was done by Diamino Benzidine (DAB) and the slides were counterstained with Mayer’s hematoxylin. PSMA IHC was also performed manually, with antigen retrieval with TRIS/EDTA, pH 9.0, dilution of 1:10, incubation time of 1 h, and detection with multimere and amplifier (Ventana, Oro Valley, AZ, USA).

### 2.7. Digital Scoring of Ki-67

Prior to the study, an MTC-specific algorithm was developed and validated for clinical purposes to allow determination of Ki-67 by artificial intelligence (unpublished for MTC; however, the same methodology was published for breast cancer [17]). On the digital whole sections, the entire tumor region was identified and marked. Preexistent thyroid epithelium and artifacts were excluded when present. The nuclear classification algorithm detected nuclei by their morphological size and form and classified these as positive or negative according to pixel color and intensity. The Ki-67 proliferation index was calculated by dividing the number of Ki-67 positive cells by the total number of cells (positive and negative) within the tumor area.

### 2.8. Scoring of Biomarkers

Each core on the TMA (three per patient) was scored per biomarker. This was performed by a thyroid pathologist (B.M.), an MTC researcher (E.J.) and a thyroid laboratory technician (B.R.). Since EZH2 staining was relatively subtle in this tumor, the degree of positive scoring was adjusted to account for the nuanced and less-pronounced staining patterns in this specific tumor type. EZH2 expression was scored as positive when at least one core was positively stained. When positive, the intensity was scored as 0 = absent, 1 = weak, 2 = moderate and 3 = prominent, and the percentage of positive tumor cells was scored negative = 0%, low = 1–9% and high ≥ 10%. PD-L1 expression was scored as positive or negative. When positive, the percentage of positivity was scored as 1 = <1%, 2 = 1–20% and 3 = >20%. For EZH2 and PD-L1, the highest score determined the final score. For PSMA and CD31, the number of PSMA- and CD31-positive microvessels was determined per core. The average number of PSMA-positive and CD31-positive vessels in the tumor was obtained to reach a final score.

### 2.9. Statistical Analysis

For the description of all patients and subgroups, frequencies were used for categorical variables, and medians (interquartile range) or means (standard deviation) for continuous data (depending on distribution). Fisher’s exact test and Mann–Whitney U evaluated differences between groups. Kaplan–Meier curves were used to evaluate differences between locoregional recurrence, distant metastasis and survival between groups. Prognostic value for biochemical cure was determined through univariate logistic regression analyses. All analyses were performed in IBM SPSS Statistics Version 29 and graphs were constructed in GraphPad Prism Version 9. A *p*-value of <0.05 was considered statistically significant.

### 2.10. Ethical Approval

This study was approved by the Medical Ethics Committee of the UMCG (register number: 10779). All study procedures were performed in accordance with the Declaration of Helsinki. Patient consent was waived since only rest-material was used, which is in line with national policy.

## 3. Results

### 3.1. Patients

Primary tumors of 64 patients were included in the study. We identified 31 (48%) females and 12 (19%) hereditary MTCs (11 MEN2A and 1 MEN2B) (Table 1). Germline RET mutations in these 12 patients were as follows: Cys618Phe (*n* = 2), Val804Leu (*n* = 2), Cys634Tyr (*n* = 2), Cys618Tyr (*n* = 1), Cys634Arg (*n* = 1), Cys634Gly (*n* = 1), Met918Tyr (*n* = 1), and unknown exact mutation (*n* = 2). Of 64 patients, 61 (95%) underwent total thyroidectomy, and 3 (5%) had a hemithyroidectomy only. Initial surgery also included central compartment dissection and lateral compartment dissection in 63 (98%) and 33 (52%) patients, respectively. Postoperative calcitonin values were available in 62 patients, and 26 (42%) were biochemically cured. During follow-up, 18 of 64 patients (28%) developed a locoregional recurrence after a median of 25 months (IQR 15–55). Only two patients with hereditary MTC developed a locoregional recurrence during follow-up. At final follow-up, 21 (33%) patients (18 sporadic and 3 hereditary MTCs) had distant metastatic disease. The median time to first distant metastasis was 16 months (IQR 2–31) after diagnosis. Distant metastasis in bone, liver, lung and brain were found in 16 (25%), 10 (16%), 9 (14%) and 2 (3%) of patients, respectively. At final follow-up (median follow-up was 66 months (IQR 34–119)), 25 (40%) of 62 patients with available calcitonin measures were biochemically cured, and 17 (27%) patients were deceased (14/17 sporadic MTCs); 10 (16%) died due to MTC progression (9/10 sporadic MTCs).

### 3.2. Morphological Features on Histopathology and IMTCGS

Results of the morphological evaluation are presented in the first column of Table 2. In these 64 MTCs, the median tumor size was 28 mm (IQR 15–40). Vascular invasion and extrathyroidal extension were present in 39 (61%) and 30 (47%) cases, respectively. Multifocality was seen in 23 (36%) cases and was associated with hereditary disease (present in 11/12 hereditary cases, *p* < 0.001). In the 64 cases, a solid growth pattern was most prevalent (42; 66%). Lobular (21; 33%), trabecular (8; 13%), cribriform (2; 3%) and follicular (3; 5%) growth patterns were less common. Spindled cell morphology in any area of the tumor was high (32; 50%). Concerning nuclear features in the tumors, we noted a high nuclear grade in 13 (20%), multinucleated cells in 25 (39%) and prominent nucleoli in 16 (25%) cases. The degree of fibrosis/amyloid deposition was highly variable between tumors. Moderate or prominent degrees of amyloid/fibrosis were observed in 42 (66%) cases, respectively. Complete tumor encapsulation was seen in only 10 (16%) cases.

**Figure 1:**
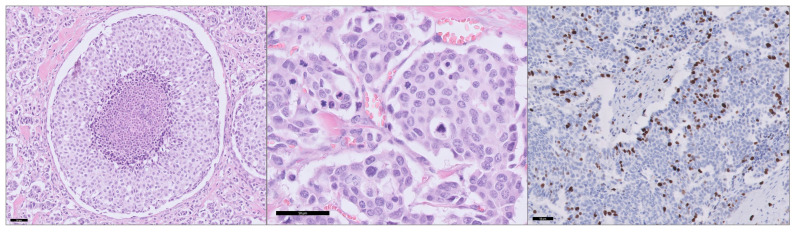
Coagulative necrosis (**left**), multiple mitoses (**middle**) and Ki-67 ≥ 5% (**right**) in an IMTCGS high-risk patient. Each scale bar per image represents a size of 50 µm.

The number of mitoses, the presence of coagulative necrosis and Ki-67 were determined and classified based on the IMTCGS. Mitoses were located in 24 patients: 1 mitosis in 13 (20%), 2 mitoses in 4 (6%), 3 mitoses in 2 (3%), 4 mitoses in 2 (3%), 5 mitoses in 1 (2%), 7 mitoses in 1 (2%), and 11 mitoses in 1 (2%) case. Coagulative necrosis was identified in 19 (30%) of all cases. Results of the Ki-67 IHC showed a median of 0.9% (IQR 0.4–2.1). Only 7 (11%) were ≥5%. Figure 1 presents example slides of coagulative necrosis, mitoses and Ki-67 ≥ 5%.

The 64 patients included in the morphological evaluation were stratified according to the IMTCGS (Table 2, columns 3 and 4), which classified our patients as low-risk (43; 67%) and high-risk MTCs (21; 33%). High-risk tumors were associated with vascular invasion (18; 86%, *p* = 0.006) and extrathyroidal extension (16; 76%, *p* = 0.001). In addition, spindled morphology was more common in high-risk tumors (15; 71%) than in low-risk MTCs (*p* = 0.032). On the contrary, we noted that a solid growth pattern was more prevalent in low-risk tumors (32; 74% vs. 10; 48%, *p* = 0.050).

### 3.3. Biomarkers with a Potential Diagnostic and Therapeutic Consequence

**Figure 2:**
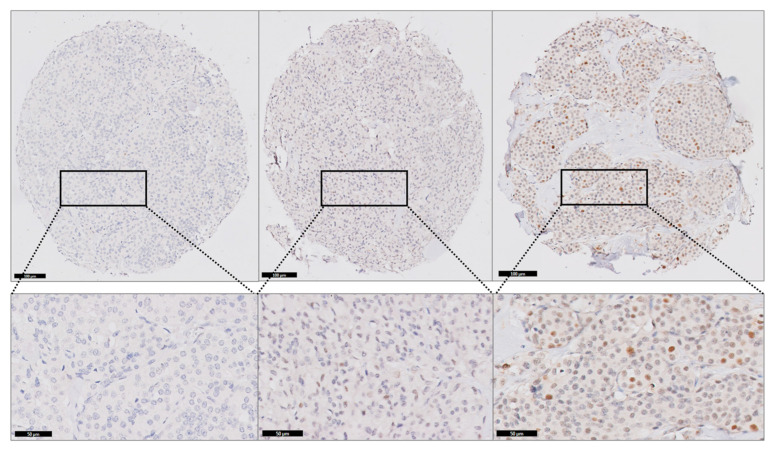
EZH2 expression in increasing percentages from left to right, left = 0% EZH2 expression (negative), middle = <10% EZH2 expression (low) and right = ≥10% EZH2 expression (high).

In total, primary tumors of 46 patients were available on the TMA (Table 3) (38 sporadic and 8 hereditary MTCs). Thirty-nine out of forty-six (85%) MTCs expressed EZH2 on IHC (33/39 sporadic MTCs), which was located in the nucleus in all cases. When positive, the intensity of EZH2 expression was scored as 1, 2 and 3 in 24 (52%), 9 (20%) and 6 (13%) cases. Overall, staining was relatively subtle, and none of the TMA cores showed more than 50% positive staining. Low EZH2 expression (<10% EZH2 expression) was seen in 30/39 (77%) of positive cases, whereas high expression (≥10% positivity) was detected in 9/39 (23%) of positive cases (8/9 sporadic MTCs). See Figure 2 for an example of negative, low and high EZH2 expression.

PD-L1 was expressed on tumor cells of ten cases in total. No expression in tumor-infiltrating immune cells was observed. 22C3 identified ten positive cases, which overlapped with the five cases identified by the SP263 staining. All positive PD-L1 cases stained <20% of tumor cells. PSMA was expressed in the neovasculature of MTCs in 45 (98%) cases but not on the epithelial cells. The median number of PSMA-positive vessels was 11 (IQR 6–19). CD31 identified the number of vessels in each tumor core, which gave a final median score of 28 vessels (IQR 17–34).

Of the tumors on the TMA, 27/34 (79%) IMTCGS low-risk MTCs were positive for EZH2. In contrast, all twelve high-risk MTCs showed EZH2 expression. This difference was not significant (*p* = 0.165). Seven of twelve (58%) high-risk tumors had EZH2 expression levels ≥ 10%, in contrast to 2/34 (6%) of low-risk MTCs (*p* < 0.001). In addition, high-risk MTCs were associated with a higher number of CD31 positive vessels (*p* = 0.021). PD-L1 expression and the number of PSMA-positive vessels did not differentiate between low- and high-risk MTCs (*p* = 0.416, *p* = 0.101, respectively).

### 3.4. Prognostic Factors

**Figure 3:**
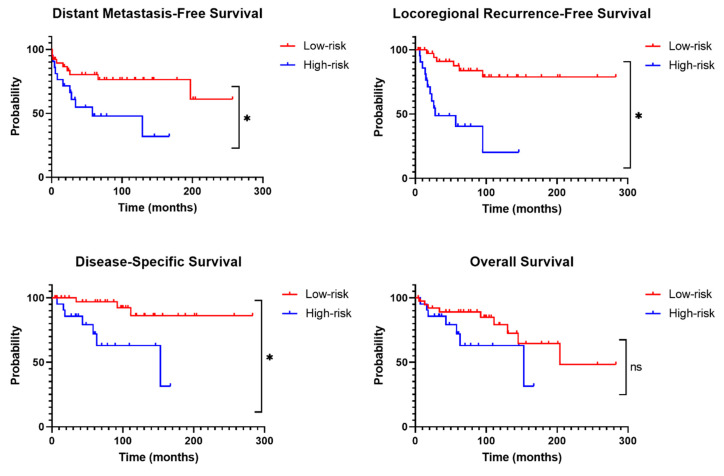
Survival curves for distant metastasis-free survival, locoregional recurrence-free survival, disease-specific survival and overall survival for low-risk and high-risk MTCs according to the IMTCGS. * = significant difference between curves (*p* < 0.05), ns = non-significant difference between curves (*p* > 0.05).

In terms of prognostic value, the high-risk MTCs were associated with lower distant metastasis-free survival (DMFS) (HR 5.651, *p* = 0.017), locoregional recurrence-free survival (LRFS) (HR 18.323, *p* < 0.001) and disease-specific survival (DSS) (HR 10.001, *p* = 0.002). In addition, classification according to the IMTCGS predicted postoperative biochemical cure (HR 0.298, *p* = 0.043), while it did not predict overall survival (OS) (HR 2.109, *p* = 0.146) or biochemical cure at last follow-up (HR 0.463, *p* = 0.181) (Figure 3).

**Figure 4:**
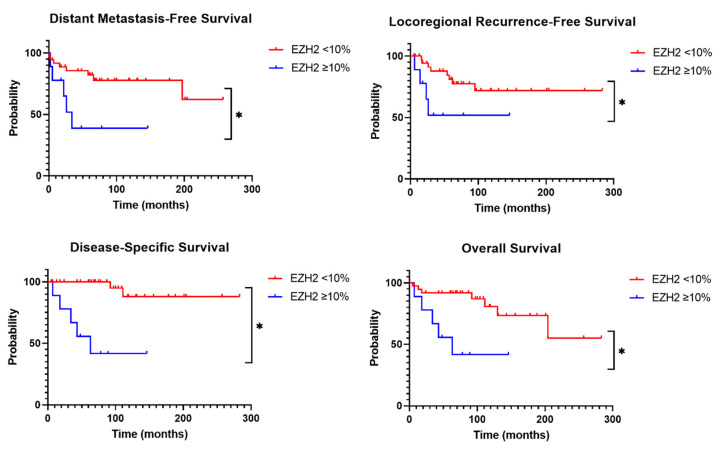
Survival curves for distant metastasis-free survival, locoregional recurrence-free survival, disease-specific survival and overall survival for EZH2 < 10% versus EZH2 ≥ 10% expression levels. * = significant difference between curves (*p* < 0.05).

Nine of 37 (24%) tumors with <10% EZH2 expression developed distant metastasis at 66 months (IQR 25–125) after primary thyroid surgery, in contrast to five of nine (56%) tumors with ≥10% EZH2 expression (at 26 months, IQR 12–63, HR 4.747 for ≥10%, *p* = 0.030)—see Figure 4. A locoregional recurrence was also more prevalent in tumors with EZH2 positivity of ≥10%, which developed after a median of 19 months (IQR 8–25), versus 44 months (IQR 19–61) (HR 4.242, *p* = 0.039). In addition, disease-specific and overall survival were worse in the presence of ≥10% EZH2 expression (HR 19.736 *p* < 0.001, HR 8.386 *p* = 0.004, respectively), compared to <10% EZH2 positivity. EZH2 positivity of ≥10% was not predictive of biochemical cure postoperatively or at final follow-up (HR 0.472 *p* = 0.388, HR 0.529 *p* = 0.417, respectively). The presence of PD-L1 expression, number of PSMA-positive vessels or number of CD31-positive vessels had no impact on patient outcomes.

## 4. Discussion

Medullary thyroid carcinoma has long been recognized as an elusive tumor with an unpredictable clinical course. Publication of the International Medullary Thyroid Cancer Grading Scheme has significantly improved baseline risk stratification of resected tumors. In this study, we validated the IMTCGS in a Dutch cohort of MTC patients and showed that the IMTCGS predicts the risk for recurrence, distant metastasis and disease-specific survival. Necrosis played a significant role in classifying patients, more so than the Ki-67 and mitotic rate. Additionally, this study is the first to explore protein expression of the epigenetic modulator EZH2, revealing relatively subtle expression in most tumors. Albeit a small cohort, relatively high expression was found to have prognostic significance, underscoring EZH2 as a promising target for future research. This finding also suggests the potential for MTC patients to participate in clinical trials exploring epigenetic therapies using EZH2 inhibitors.

In this study of 64 patients, 67% and 33% were stratified as low- and high-risk, respectively, according to the IMTCGS. Most high-risk classifications were based on coagulative necrosis, while sole presence of a high mitotic index or Ki-67 classified only two extra high-risk patients. Since the original publication of the IMTCGS by Xu et al. in 2021, to our knowledge, three validation studies have been published [5,18,19,20]. The proportion of high-risk patients varied between 18–32% [18,19,20], which is comparable to our cohort. According to Xu et al., the prognostic capacity of necrosis was strong on its own, but the Ki-67 and mitotic index were added to the grading scheme to improve the classification even further [5]. Necrosis is relatively easy to identify for a trained pathologist, while mitoses are more difficult and time-consuming to identify, and Ki-67 requires staining in addition to a validated scoring technique. The mitotic rate is also the most difficult to reproduce, as shown in an interobserver reproducibility study [21]. Our study provides reassurance to pathologists, knowing that as long as they accurately identify the presence of coagulative necrosis, most patients can be classified according to the IMTCGS, ensuring adequate risk stratification of the majority of MTC patients.

In terms of prognostic value, we show that the IMTCGS was able to accurately predict the locoregional recurrence-free survival, distant metastasis-free survival and disease-specific survival in a Dutch cohort of MTC patients, which is in line with validation studies in other populations [18,19,20]. While overall survival was lower in the high-risk group, it was not significant in our study. This contrasts with Xu’s study but is in line with the validation study by Vissio et al. and may be a result of smaller cohorts than the original publication [5,18].

Lymph node metastases at diagnosis were not associated with low- or high-risk disease in our study, confirming similar results in the studies by Vissio et al. and Rai et al. [18,20]. This fact potentially shows that, when lymph node metastases are accurately managed in treatment, their sole presence does not have to impact the ultimate prognosis, as long as the primary tumor characteristics are favorable. On the contrary, extrathyroidal extension and vascular invasion were more prevalent in high-risk IMTCGS patients, and identify more aggressive tumors, which is concordant with previous literature [22,23]. Most other morphological features we evaluated in this study were not associated with low- or high-risk IMTCGS. However, we did identify a near-significant (*p* = 0.050) association between the presence of a solid growth pattern and low-risk tumors. A solid growth pattern, usually in nests or sheets, is often used to describe the classical MTC growth pattern. Moura et al. demonstrated an association between high-risk somatic M918T mutation or A883P *RET* mutation and classical growth type, but also between the absence of a *RET* or *RAS* mutation and this growth type, showing considerable heterogeneity in the presence of a growth type and the associated clinical behavior [4]. Spindled morphology was seen in a relatively high number of patients and was associated with the high-risk IMTCGS category, portending a worse prognosis. Moura et al. described spindled cell type in only 6% of cases, possibly because they described the dominant growth type, instead of any observed growth pattern like we did, with an intermediate prognosis [4]. Fuchs et al. showed <50% and ≥50% spindled morphology in 18% and 82% of cases, respectively; no association with prognosis was identified [24].

While the IMTCGS is an accurate prognosticator, its parameters have no therapeutic value. In this study, we also evaluated the prevalence of EZH2, PSMA, and PD-L1, which have theranostic and/or therapeutic potential in addition to possible prognostic value. To our knowledge, this is the first time EZH2 protein expression has been evaluated in an MTC cohort. EZH2 expression was identified in 85% of 46 cases. Interestingly, EZH2 prevalence in more than 10% of the examined tissue (seen in 20% of cases) has prognostic power with lower distant metastasis-free survival, locoregional recurrence-free survival, disease-specific survival and overall survival. Our data are in line with the study by Sponziello et al., which reported that high epigenetic-related gene expression of EZH2 portends a worse prognosis with higher occurrence of metastasis, persistent disease and disease-specific death [16].

Over the last few decades, EZH2 has gained attention in the oncological field. In breast cancer, EZH2 is now an important marker of aggressive behavior, with strong evidence for transcriptional repression and neoplastic transformation in cell-line studies [15], and higher expression in metastatic lesions than in primary lesions [14]. A study in thyroid cancer patients identified absent EZH2 expression in normal thyroid tissue and differentiated thyroid cancer, but presence in poorly differentiated (PDTC) and anaplastic thyroid cancers. In PDTC, high expression was associated with an increased risk for distant metastasis [12]. Studies in neuroendocrine tumors (NETs) show similar associations between overexpression and disease aggressivity [13,25]. In a cell-line study of pancreatic NETs, inhibition of EZH2 led to lower cell viability and reduced proliferative activity [13]. Multiple compounds with different mechanisms of action against EZH2 have been developed or are being developed. Probably the most potent inhibitor so far is tazemetostat, which is under evaluation in several clinical trials that show promising preliminary results. The trials include mostly hematological diseases, but some also focus on other advanced solid tumors, showing potential for patients with other cancer types, such as MTC [10,11].

Expression of PSMA was seen in the neovasculature of nearly all evaluated MTC tumors. However, epithelial expression was not identified in any case. This is in line with the study by Lodewijk et al., although we were not able to confirm that high PSMA expression predicts the locoregional recurrence risk [26]. PSMA did not have any prognostic value in our study. Nevertheless, high PSMA expression in the vessels may explain case reports of incidental MTC findings after uptake of PSMA-labeled PET/CT imaging [27]. In addition, high expression in comparison to healthy tissue may even indicate potential for ^177^Lu-PSMA-617 radionuclide therapy, although more research is necessary before it may be initiated [28]. PD-L1 was seen in very few cases and did not influence patients’ outcomes. This is in line with previous histological studies, which show that PD-L1 ranges between 14–22% positivity [29,30]. PD-L1 expression, therefore, seems to be low in MTC, which may explain why little is expected of PD-L1 checkpoint inhibitors for MTC treatment, in contrast to in anaplastic thyroid cancer.

Although a significant cohort for such a rare disease, the present study may have been limited by the sample size and the retrospective nature. For the evaluation of biomarkers, TMA cores were stained and scored, instead of whole sections, which may have impacted scores if cores were incidentally taken from a biomarker-rich or biomarker-poor area. Moreover, both the morphological features and biomarkers (except for Ki-67) were scored manually, creating potential interobserver variability. However, this is inherent to the work of a pathologist, and we were able to validate the IMTCGS despite this. By using a consistent method throughout all scoring sessions, we aimed to prevent potential bias.

Future efforts should focus on confirming EZH2 expression in a larger cohort of MTC patients, correlating protein expression with gene expression patterns, and aiming to evaluate EZH2 expression in metastatic lesions as well. Additionally, methylation studies should be performed to elucidate the potential mechanisms and downstream effects of EZH2 overexpression in MTC. After confirmation of its prognostic power, scoring should be standardized to allow for reproducibility. After initial testing in cell-line studies, selected patients may be eligible for inclusion in clinical trials for EZH2 inhibitors, with the ultimate aim of improving therapeutic options and clinical outcomes for patients with this rare disease.

## 5. Conclusions

This study adds evidence to the prognostic value of the IMTCGS and identifies EZH2 as a novel biomarker and prognosticator in MTC patients. The potential of EZH2 as a therapeutic target highlights its relevance and warrants further exploration in future studies, including larger cohorts.

## Figures and Tables

**Table 1:** Patient characteristics.

	All Patients *n* = 64 *n* (%) or Median (IQR)	Low-Risk MTC *n* = 43 *n* (%) or Median (IQR)	High-Risk MTC *n* = 21 *n* (%) or Median (IQR)	Low vs. High *p*-Value
Sex				0.035
Male	33 (52)	18 (42)	15 (71)	
Female	31 (48)	25 (58)	6 (29)	
Type of MTC				1.000
Sporadic	52 (81)	35 (81)	17 (81)	
Hereditary	12 (19)	8 (19)	4 (19)	
T-stage				0.803
T1	22 (34)	16 (37)	6 (29)	
T2	25 (39)	16 (37)	9 (43)	
T3	13 (20)	9 (21)	4 (19)	
T4	4 (6)	2 (5)	2 (10)	
N-stage				0.351
N0	18 (28)	14 (33)	4 (19)	
N1a	12 (19)	9 (21)	3 (14)	
N1b	34 (53)	20 (47)	14 (67)	
M-stage				0.460
M0	55 (86)	38 (88)	17 (81)	
M1	9 (14)	5 (12)	4 (19)	
TNM				0.324
I	8 (13)	6 (14)	2 (10)	
II	10 (16)	8 (19)	2 (10)	
III	8 (13)	7 (16)	1 (5)	
IV	38 (59)	22 (51)	16 (76)	

Percentage totals may not sum to 100% due to rounding. Abbreviations: IQR = interquartile range, MTC = medullary thyroid carcinoma.

**Table 2:** Morphological features.

	All Patients *n* = 64 *n* (%) or Median (IQR)	Low-Risk MTC *n* = 43 *n* (%) or Median (IQR)	High-Risk MTC *n* = 21 *n* (%) or Median (IQR)	Low vs. High *p*-Value
Tumor size in mm	28 (15–40)	27 (13–40)	30 (17–43)	0.529
Vascular invasion	39 (61)	21 (49)	18 (86)	0.006
Perineural growth	5 (8)	2 (5)	3 (14)	0.320
Extrathyroidal extension	30 (47)	14 (33)	16 (76)	0.001
Positive resection margin	27 (42)	15 (35)	12 (57)	0.111
Multifocality	23 (36)	15 (35)	8 (38)	1.000
Bilaterality *	16 (26)	11 (26)	5 (26)	1.000
Growth pattern				
Solid	42 (66)	32 (74)	10 (48)	0.050
Lobular	21 (33)	11 (26)	10 (48)	0.095
Trabecular	8 (13)	4 (9)	4 (19)	0.422
Cribriform	2 (3)	1 (2)	1 (5)	1.000
Follicular	3 (5)	2 (5)	1 (5)	1.000
Cell shape				
Round	16 (25)	9 (21)	7 (33)	0.360
Polygonal	45 (70)	28 (65)	17 (81)	0.251
Spindled	32 (50)	17 (40)	15 (71)	0.032
Hyperchromatic nuclei	13 (20)	9 (21)	4 (19)	1.000
Nuclear grade				0.785
Low	22 (34)	16 (37)	6 (29)	
Intermediate	29 (45)	18 (42)	11 (52)	
High	13 (20)	9 (21)	4 (19)	
Multinucleated cells	25 (39)	15 (35)	10 (48)	0.416
Prominent nucleoli	16 (25)	11 (26)	5 (24)	1.000
Oncocytic variant	17 (27)	11 (26)	6 (29)	1.000
Clear-cell variant	14 (22)	11 (26)	3 (14)	0.356
Fibrosis/amyloid				0.269
None–mild	22 (34)	17 (40)	5 (24)	
Moderate–prominent	42 (66)	26 (60)	16 (76)	
Microcalcifications	19 (30)	13 (30)	6 (29)	1.000
Encapsulation				0.242
None	32 (50)	21 (49)	11 (52)	
Partially	22 (34)	13 (30)	9 (43)	
Completely	10 (16)	9 (21)	1 (5)	
Tumor necrosis	19 (30)	0 (0)	19 (90)	<0.001
Mitotic count ≥ 5	3 (5)	0 (0)	3 (14)	0.032
Ki-67 ≥ 5% **	7 (11)	0 (0)	7 (35)	<0.001

Percentage totals may not sum to 100% due to rounding. * Bilaterality could not be determined in three cases where no total thyroidectomy was performed. ** Ki-67 could not be determined in 1 patient, but this patient could still be classified according to the IMTCGS due to the presence of necrosis in these patients. Abbreviations: IQR = interquartile range, MTC = medullary thyroid carcinoma.

**Table 3:** Biomarker expression on immunohistochemistry.

Biomarker	Patients *n* = 46 *n* (%) or Median (IQR)	Low-Risk MTC *n* = 34 *n* (%) or Median (IQR)	High-Risk MTC *n* = 12 *n* (%) or Median (IQR)	Low vs. High *p*-Value
EZH2 intensity				0.022
0	7 (15)	7 (21)	0 (0)	
1	24 (52)	20 (59)	4 (33)	
2	9 (20)	4 (12)	5 (42)	
3	6 (13)	3 (9)	3 (25)	
EZH2% positivity				0.165
Negative 0%	7 (15)	7 (21)	0 (0)	
Low 1–9%	30 (65)	25 (74)	5 (42)	
High ≥ 10%	9 (20)	2 (6)	7 (58)	
PD-L1 combined score	10 (22)	6 (18)	4 (33)	0.416
Clone 22C3	10 (22)	6 (18)	4 (33)	
Clone SP263	5 (11)	3 (9)	2 (17)	
PSMA average number of positive vessels	11 (6–19)	10 (5–17)	15 (7–29)	0.101
CD31 average number of positive vessels	28 (17–34)	22 (14–29)	33 (24–47)	0.021

Percentage totals may not sum to 100% due to rounding. Abbreviations: IQR = interquartile range, MTC = medullary thyroid carcinoma, EZH2 = enhancer of zeste homolog 2, PD-L1 = programmed death-ligand 1, PSMA = prostate-specific membrane antigen.

## Data Availability

The datasets presented in this article are not readily available because the data are part of ongoing research. Requests to access the datasets should be directed to the corresponding author.
